# COPA3 peptide supplementation alleviates the heat stress of chicken fibroblasts

**DOI:** 10.3389/fvets.2023.985040

**Published:** 2023-02-24

**Authors:** Sharif Hasan Siddiqui, Mousumee Khan, Jinryong Park, Jeongeun Lee, Hosung Choe, Kwanseob Shim, Darae Kang

**Affiliations:** ^1^Center for Musculoskeletal Research, School of Medicine and Dentistry, University of Rochester Medical Center, Rochester, NY, United States; ^2^Karmanos Cancer Institute, Wayne State University, Detroit, MI, United States; ^3^Department of Biomedical Sciences and Institute for Medical Science, Jeonbuk National University Medical School, Jeonju, Republic of Korea; ^4^Department of Animal Biotechnology, Jeonbuk National University, Jeonju, Republic of Korea; ^5^Department of Stem Cell and Regenerative Biotechnology, Konkuk University, Seoul, Republic of Korea; ^6^3D Tissue Culture Research Center, Konkuk University, Seoul, Republic of Korea; ^7^Department of Agricultural Convergence Technology, Jeonbuk National University, Jeonju, Republic of Korea

**Keywords:** heat stress, fibroblast, COPA3, viability, proliferation, heat tolerance

## Abstract

Heat stress inhibits cellular proliferation and differentiation through the production of reactive oxygen species. Under stress conditions, antioxidant drugs promote stable cellular function by reducing the stress level. We sought to demonstrate 9-mer disulfide dimer peptide (COPA3) supplementation stabilizes fibroblast proliferation and differentiation even under heat stress conditions. In our study, fibroblasts were assigned to two different groups based on the temperature, like 38°C group presented as Control - and 43°C group presented as Heat Stress-. Each group was subdivided into two groups depending upon COPA3 treatment, like 38°C + COPA3 group symbolized Control+ and the 43°C + COPA3 group symbolized as Heat Stress+. Heat stress was observed to decrease the fibroblast viability and function and resulted in alterations in the fibroblast shape and cytoskeleton structure. In contrast, COPA3 stabilized the fibroblast viability, shape, and function. Moreover, heat stress and COPA3 were found to have opposite actions with respect to energy production, which facilitates the stabilization of cellular functions by increasing the heat tolerance capacity. The gene expression levels of antioxidant and heat shock proteins were higher after heat stress. Additionally, heat stress promotes the mitogen-activated protein kinase/ extracellular signal-regulated kinase–nuclear factor erythroid 2-related factor 2 (MAPK/ERK-Nrf2). COPA3 maintained the MAPK/ERK-Nrf2 gene expressions that promote stable fibroblast proliferation, and differentiation as well as suppress apoptosis. These findings suggest that COPA3 supplementation increases the heat tolerance capacity, viability, and functional activity of fibroblasts.

## Introduction

Heat stress is such a temperature where cell or organism losses normal physiological and molecular function, including metabolic activity (1), protein stability (2), immunoglobulin (3), cytoskeleton function (4), and eventually overall loss of cell function (5). Moreover, heat stress is also responsible for the imbalance production and accumulation of reactive oxygen species (ROS) (6) produced by oxygen metabolism. ROS, the by-products of cellular respiration, are continuously produced in all aerobic organisms (7). It has been reported that ROS are produced by different cellular processes and stimulation (8). The imbalance of ROS production is an important cause of the oxidative stress (6). Unfortunately, heat stress is one of the factors to induces oxidative stress by unbalancing ROS production and accumulation in cells (9). Additionally, heat stress generates a lot of problems in cell survival. The stressed cells build a defense system that stimulates the enzymatic elements superoxide dismutase (SOD) and catalase (CAT) to shield themselves from the oxidative stress-induced cell damage (10). Heat stress induces protein misfolding and denatures (11). However, the heat shock proteins (HSPs) protect the cell by preventing protein misfolding and aggregation (12). Although, HSPs expression depends on different organs and heat intensity (13). During stress, the glutathione S-transferase (GST) gene family plays an important role in the cellular life processes and controls toxification and detoxification by conjugation of glutathione with numerous pharmaceuticals and environmental pollutants (14). GST gene family is categorized into six subfamilies, namely alpha (GSTA), mu (GSTM), omega (GSTO), pi (GSTP), theta (GSTT), and zeta (GSTZ), and contains a total of 16 genes (14). Heat shock proteins (HSPs) are synthesized by cells to protect themselves by preventing protein denaturation and restoring the folded state of denatured proteins from heat stress (15). The oxidative stress level in cells, after the activation of different pathways and substrates, can be measured by analyzing biochemical parameters such as lactate dehydrogenase (LDH) and nitric oxide (NO) (16, 17). The NO and LDH level both increases during heat stress and both are indicating a stress condition (18, 19).

The mitogen-activated protein kinase (MAPK), one of the most crucial signaling pathways, stimulates eukaryotic cellular processes such as cell proliferation, differentiation, apoptosis, and stress response (20). MAPK acts as the central signaling element that transmits cellular signals from the extracellular environment to intercellular targets (21, 22). MAPK is classified into extracellular signal-regulated kinase (ERK), Jun kinase (JNK/SAPK), and p38 MAPK, which regulate cell function-related genes by controlling the cell cycle (23). The JNK and p38 MAPK signaling pathways are closely related to cell proliferation and differentiation and play a crucial role in the cell signal transduction network (24–26). The MAPK/ERK signaling pathway is the core signaling cascade that regulates cell division, growth, development, and survival (27). The nuclear factor erythroid 2–related factor 2 (Nrf2), a key regulator of cellular resistance to oxidative stress, regulates oxidative stress response by controlling physiological and pathophysiological gene expression (28).

The insect peptide COPA3 is a 9-mer disulfide dimer peptide (LLCIALRKK-NH2, D-form) synthesized from coprisin which extracted from Korean dung beetle (*Copris tripartitus*) (29). This coprisin has 80 amino acids which molecular weight is 8.6 kDa (30). COPA3 contains two monomers linked by a disulfide bond (31). Previous study reported that the disulfide bond maintains oxidant levels by adjusting the protein's function (32). Moreover, the anti-inflammatory, antimicrobial, anti-neuronal apoptotic and anti-cancer effects of COPA3 are well documented (29, 31, 33, 34). Moreover, COPA3 prevents neuronal cell apoptosis through the degradation of the p27Ki1 protein (29). However, the influence of COPA3 on heat stress induced oxidative stress in broiler's (meat type chicken) skeletal muscle fibroblasts, as well as the function of COPA3 on cell against oxidative stress and how regulates the MAPK/ERK-Nrf2 gene expression, are not understood. The present study was aimed at investigating the effect of COPA3 on the proliferation and differentiation of broiler skeletal muscle fibroblasts under oxidative stress through the regulation of gene expression of the MAPK/ERK-Nrf2 related genes (35). Fibroblasts are common cells found in connective tissue which maintain the structural integrity of tissue or organ by secreting extracellular matrix (36). We hypothesize the COPA3 will help broiler chicken production by reducing heat stress severity in *in-vivo* studies. Our data suggested that the COPA3 suppresses the effect of heat stress on fibroblasts homeostasis by regulating the MAPK/ERK-Nrf2 related gene expression.

## Materials and methods

### Reagents

For cell culture, we used high-glucose Dulbecco's modified Eagle medium (DMEM; Gibco, Cat. No. 11965092), fetal bovine serum (FBS; Gibco, Cat. No. 10270106), phosphate-buffered saline (PBS; LPS solution, Cat. No. CBP007B), and 100 × penicillin–streptomycin (10,000 U/mL; Invitrogen, NY, USA; Cat. No. 15140-122).

### Fibroblasts culture and experimental design

**Figure 1:**
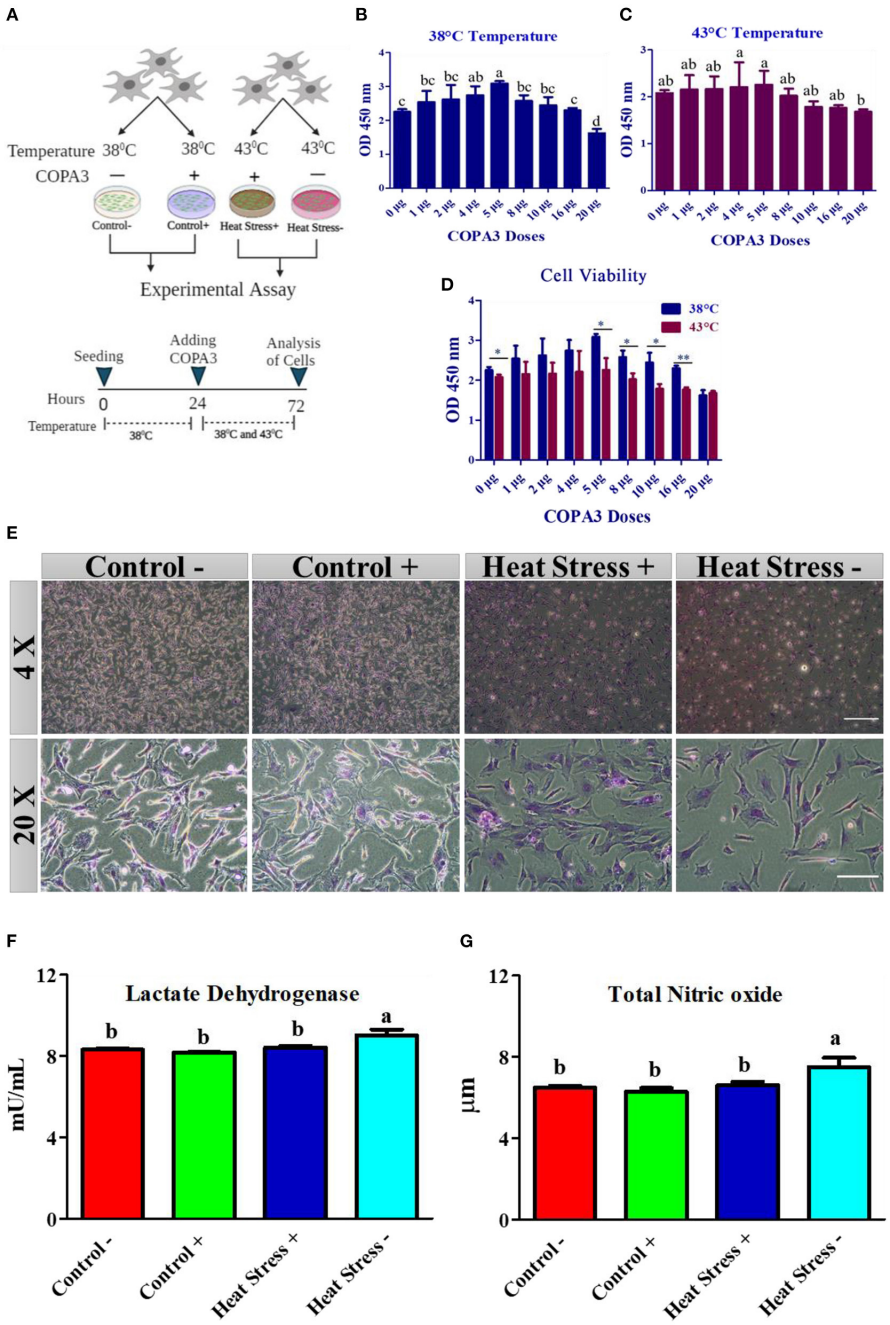
COPA3 protects the stressed fibroblasts' morphology by increasing heat tolerance. **(A)** Model experimental strategy; 38°C and 43°C temperatures indicate the control and the heat stress group, respectively; each group was subdivided into two groups based on COPA3 treatment. **(B)** Survival rates of fibroblasts at 38°C obtained using the CCK-8 assay; data represent mean ± SE; ^a−*d*^different letters indicate significant difference in mean fibroblasts in 38°C (*P* < 0.05). **(C)** Survival rate of fibroblasts at 43°C temperature obtained using the CCK-8 assay; data represent mean ±SE; ^a, b^different letters indicate significant difference in the mean number of fibroblasts at 43°C (*P* < 0.05). **(D)** Comparative survival rates of the fibroblasts at 38°C and 43°C obtained using the CCK-8 assay; data represent mean ± SE; *P < 0.05, and **P < 0.001. **(E)** Giemsa staining showing the morphology of the fibroblasts at 4 × and 20 × microscopic focus; scale bar = 100 μm and 20 μm, respectively. **(F)** Concentration of lactate dehydrogenase (LDH); data represent mean ±SE; ^a, b^different letters indicate significant difference in different groups (*P* < 0.05). **(G)** Concentration of nitric oxide (NO); data represent mean ± SE; ^a, b^different letters indicate significant difference in different groups (*P* < 0.05). Groups are distinguished by temperatures and COPA3 treatment, that is, fibroblasts at 38°C (Control- group), 38°C with COPA3 treatment (Control+ group), 43°C with COPA3 treatment (Heat Stress+ group), and 43°C temperature (Heat Stress- group). Each experiment was conducted in four biological replicates (*n* = 4).

Broiler fibroblasts were isolated earlier in the Department of Animal Biotechnology, Jeonbuk National University which were previously described (37). The Rural Development Administration (RDA), Korea, donated the COPA3 used in this study. The purity of our experimental COPA3 was 97%. We have used acidified distilled water (0.01% acetic acid) as a solvent to dissolve the COPA3 powder for our experiment (31). The cells were cultured in 75 mm cell culture flasks containing DMEM (Life Technologies, Grand Island, NY) with 10% fetal bovine serum (FBS) (Sigma-Aldrich, St. Louis, MO), 5% horse serum (Sigma-Aldrich, St. Louis, MO), and 1% antibiotic/penicillin–streptomycin (Life Technologies, Grand Island, NY) at 38°C in a humidified incubator with 5% CO_2_/95% O_2_. The cells were cultured using two different incubators which for control and heat stress groups as 38 and 43°C with 5% CO2, respectively. The cells were cultures in 75 mm cell culture flasks containing DMEM (Life Technologies, Grand Island, NY) with 10% fetal bovine serum (FBS) (Sigma-Aldrich, St. Louis, MO), 5% horse serum (Sigma-Aldrich, St. Louis, MO), and 1% antibiotic/penicillin–streptomycin (Life Technologies, Grand Island, NY) at 38°C. At the exponential growth phase (approximately 80% confluence), the cells were subcultured to increase the number of cells and passage number. After obtaining a sufficient number of cells, we seeded the cells for heat stress treatment and COPA3 supplementation. Earlier, the cells were seeded at 38°C for 24 h for cell adaptation. Then, this study was categorized into two groups based on the incubation temperature: 38°C and 43°C as control and heat stress, respectively. Both groups were subdivided based on the addition of COPA3 as follows: the 38°C group denoted as Control -, 38°C + COPA3 group denoted as Control+, 43°C group denoted as Heat Stress-, and the 43°C + COPA3 group denoted as Heat Stress+ (Figure 1A). In this study, we selected 38°C as a control temperature and 43°C as a stress temperature for chicken cell (38).

### COPA3 dose selection based on fibroblast viability

Fibroblast viability analyses were conducted to select the optimal dose of COPA3 at the incubation temperatures, and a dose that induced a relatively high viability was selected for further experiments. We also performed fibroblast viability analysis to determine whether COPA3 maintains fibroblast viability under heat stress. A total of 10,000 cells/well were seeded into a 96-well plate, and after 24 h of incubation at the specified temperature, COPA3 was added at the recommended dose. Cell viability was analyzed after 48 h of incubation using the CCK-8 assay (Dojindo, Cat. No. CK04). CCK-8 reagent (10 μL) was added to each well and incubated at the specified temperature for 4 h, and cell viability was measured using a microplate reader at 450 nm absorbance. Each assay was replicated four times.

### Giemsa staining

The cells (1×10^6^) were seeded into a 6-well plate. After heat stress, the media was removed and the cells were fixed for 5 min in methanol, and then dried completely. Giemsa stain (Sigma, MA, USA) was diluted with deionized water (1:20), and the samples were stained with Giemsa stain for 30 min. The stain was washed with running tap water and air dried, and the images were observed using an inverter microscope (40×; CKX53, Olympus, Tokyo, Japan).

### Measurement of nitrite and nitrate concentration and LDH activity

After incubation period of heat stress, the fibroblasts were harvested and stored immediately at −80°C for further analyses. To measure LDH activity, the frozen cells (1 × 10^6^ cells) were homogenized with PBS by vortexing. LDH activity was measured using a colorimetric lactate dehydrogenase assay kit according to the manufacturer's instructions (Abnova, Walnut, CA. # cat- KA0878). The concentrations of nitrite and nitrate were determined using a colorimetric oxiselect™ *in vitro* nitric oxide (nitrite/nitrate) assay kit (Cell Biolabs, Inc., San Diego, USA). # Cat- STA-802) according to the manufacturer's instructions.

### Cell migration assay

Fibroblast migration was measured at different temperatures after COPA3 treatment. A total of 1 × 10^6^ cells were seeded in each well of a 6-well plate. After attaining 90% confluence, the cells were wounded by scratching using a sterile pipette tip, and impaired and dead cells were washed out by 1X PBS. Complete medium (89% DMEM medium + 10% FBS + 1% penicillin-streptomycin) (2 mL) was added to each well. The cells were subjected to different temperatures (38°C and 43°C) and COPA3 peptide treatment (5 μg/mL) and observed 12 h post incubation (12 hpi) and 24 hpi. After incubating for the respective time periods, images were taken using an inverter microscope (Olympus). The scratched area was measured by observing the images using ImageJ software (NIH, Bethesda, MD, USA). After quantification of the wounded area, the 0 hpi wounded area was normalized to 10,000 μm2 area. The wounded cell values were then compared with the normalized values at 12 hpi and 24 hpi for each group (39).

### Coomassie brilliant blue staining assay

The fibroblasts morphology was measured at different temperatures after COPA3 treatment. A total of 1×10^6^ cells were seeded in each well of a 6-well plate. After attaining 50% confluence, the cells were subjected to different temperatures (38 and 43°C) and COPA3 peptide (5 μg/mL) treatment. After 48 h of incubation, the media was removed and the cells were fixed using 100% methanol for 20 min. The cells were then aspirated with distilled water and Coomassie brilliant blue was added for 30 min. Next, the cells were washed three times with distilled water and dipped in xylene for 15 min to differentiate the cytoskeleton from the background. Finally, the images were observed using an inverter microscope (Olympus).

### Oil red O staining

To observe lipid accumulation in the fibroblasts at different treatments Oil Red O stain was used. Fibroblasts were cultured in a 6-well plate for oil red staining under two different culture medium conditions. One culture medium contained DMEM with 10% FBS and 1% penicillin-streptomycin and was termed as the proliferation medium, and the other contained DMEM with 1% penicillin-streptomycin and was termed as the differentiation medium. After the recommended incubation time, the cells were treated with COPA3, gently washed with PBS, and fixed with 10% formalin for 1 h at room temperature (24°C). Formalin was removed and washed with distilled water, and the cells were then incubated with 60% isopropanol for 5 min at room temperature. Isopropanol was discarded and the cells were stained with Oil Red O solution (0.5% Oil Red O in isopropanol). The Oil Red O solution was discarded, and the samples were washed with water. Hematoxylin was added to the cells; the cells were incubated for 1 min, washed with water, and observed under a microscope (Olympus). Lipid content was measured by direct extraction of Oil Red O from the stained cells using isopropanol and absorbance at 492 nm was determined using a microplate reader (Multiskan GO Microplate Spectrophotometer, Thermo Scientific, MA).

### Measurement of cellular ATP concentration

Cellular ATP concentrations were measured using a colorimetric ATP assay kit (Abnova, Walnut, CA. # cat- KA0806) according to the manufacturer's instructions. Dissociated fibroblasts were lysed and centrifuged at 12,000 × g for 5 min at 4°C. The supernatant (40 μL) was mixed with 10 μL of ATP Assay Buffer in a 96-well plate. Reaction mixture (50 μL) was added to the standard and test samples. This mixture was incubated for 30 min at room temperature, and absorbance was measured at 570 nm using a microplate reader (Thermo Fisher Scientific, Waltham, MA, USA). ATP concentration was measured in μmol/g. Each independent experiment was performed in triplicates.

### Measurement of G6PDH activity

The G6PDH level was measured using a colorimetric assay kit (Cell Biolabs, Inc., San Diego, CA, USA) according to the manufacturer's instructions. The assay kit included a G6PDH enzyme and had a detection sensitivity limit of ~1 mU/mL. A total of 1×10^7^ cells were centrifuged at 1,000 × g for 10 min. The culture medium was aspirated carefully and washed once with cold PBS.

The cells were homogenized in 1 mL cold lysis buffer by vortexing to prepare the cell suspension. The cell suspension and the reagents were mixed in a 96-well plate. The plate was then incubated at 37°C for 15 min. The absorbance of the samples was measured at 450 nm using a spectrophotometer (Multiskan GO Microplate Spectrophotometer, Thermo Scientific, MA).

### Live and dead cell image acquisition

Live and dead cell images were obtained using a Live/Dead^®^ Cell Imaging kit (488/570; R37601; Thermo Fisher Scientific) according to the manufacturer's instructions. Hydrolyzes the cell membrane permeable compounds that convert to the fluorescent anion calcein, thus detecting live cells. The dead cells were detected using ethidium homodimer (EthD-1) that stains the dead cells, which are impermeable to the dye, due to a compromised cell membrane. Images were obtained using a confocal laser scanning microscope (LSM 700; Carl Zeiss Microscopy Co., Ltd., Tokyo, Japan).

### Crystal violet staining

Fibroblasts were plated at a concentration of 1 x 10^5^ cells/well in six well plate for 24 h. Then add insect peptide COPA3 and was incubated for 48 h with respective temperature. After treatment, the medium was removed and washed with PBS. The cells were fixed with methanol and acetic acid (3:1) for 5 min at room temperature (25°C). Removed fixing reagent and add 0.5% crystal violet (Sigma) for 15 min at room temperature (25°C), then wash with running tap water. Take the image for the visualization of cells. Cells were then incubated with 10% acetic acid for 20 min with shaking. All extracted 96 well plates were measured at 590 nm (Multiskan GO Microplate Spectrophotometer, Thermo Scientific, MA) to count the cells and calculate cells percentages. Each experiment was examined through triplicates.

### Oxidative stressed and COPA3 treated fibroblasts mRNA expression

Total RNA was extracted from the fibroblasts using TRIzol reagent (Invitrogen, USA) according to the manufacturer's instructions. The volume and the purity of the extracted RNA were calculated using a NanoDrop spectrometer (Invitrogen, NY, USA) at 260 nm and 260/280 nm, respectively. cDNA was synthesized from RNA by reverse transcription using the iScript cDNA synthesis kit (BIO-RAD, USA) according to the manufacturer's instructions. The synthesized cDNA was amplified using SYBR Green Supermix (Bio-Rad, USA) on a CFX96 Real-Time PCR Detection System (Bio-Rad, USA), as described previously (40, 41). Chicken-specific primers for the targeted genes (Supplementary Table 1) were used to study the relationship between heat stress and the MAPK/ERK-Nrf2. Relative mRNA fold changes were determined using the 2^−ΔΔCt^ method (42). The cycle threshold (CT) value of GAPDH was used to normalize the CT value of the targeted genes.

### Statistical analysis

All data were analyzed using SAS version 9.4 (SAS Institute Inc., Cary, NC, USA) and R version 10.1.0 (CA, USA). The fold change in the mRNA expression, colorimetric data, and quantitative data obtained from the images of the four different groups were analyzed by one-way analysis of variance (ANOVA) followed by Duncan's multiple range test. The t-test was used to analyze the effects of the different doses of COPA3 at the two different temperatures. The Pearson correlation coefficient, principal component analysis, clustering analysis, and circular plot analysis were performed using R software (version 4.1.0) using different packages such as corrplot, GGally, factoextra, ggfortify, gplots, psych, ggplot2, and circlize. The values *P* < 0.05 and *P* < 0.01 represented statistical significance.

## Results

### COPA3 regulates the homeostasis of cell morphology and stress level

To explore the role of COPA3 in antioxidant response, we optimized the COPA3 dose in primary fibroblasts by administering different doses of COPA3 at two different temperatures (38°C and 43°C). After 24 h of cell seeding in two 96-well plates, we added COPA3 and incubated the plates at 38°C and 43°C, respectively, for 48 h (Figure 1A). Significant cell proliferation was found in plates containing 5 μg/mL COPA3 incubated at 38°C and 4 and 5 μg/mL COPA3 incubated at 43°C compared to plates containing other doses of COPA3 (Figures 1B, C). Significant higher cell proliferation was observed at 0, 5, 8, 10, and 16 μg/mL at 38°C compared to those at 43°C (Figure 1D). We selected the dose 5 μg/mL COPA3 for further study, based on the aforementioned results. Cellular morphology was found to be different in the Heat Stress- (43°C + without COPA3) group when compared to that in the other groups. No cellular morphological changes were observed in the Control- (38°C + without COPA3), Control+ (38°C + COPA3), and the Heat Stress+ (43°C + COPA3) groups (Figure 1E). The levels of lactate dehydrogenase (LDH), an enzyme that affects cellular respiration, were significantly higher in the Heat Stress- group than in the other groups (Figure 1F). The total nitric oxide (NOx) level was found to be significantly higher in the Heat Stress- group than in the other groups (Figure 1G). No significant changes in the NOx and LDH levels were observed in the Control-, Control+, and Heat Stress+ groups. The amount of nitrate was found to be significantly different between the Control+ and Heat Stress- groups (Supplementary Figure 1A). No significant difference in nitrite levels were observed among the different groups (Supplementary Figure 1B). In this study, these results indicate that COPA3 shows antioxidant activity without changing the cellular morphology.

#### COPA3 maintains viability and function of fibroblasts under heat stress

**Figure 2:**
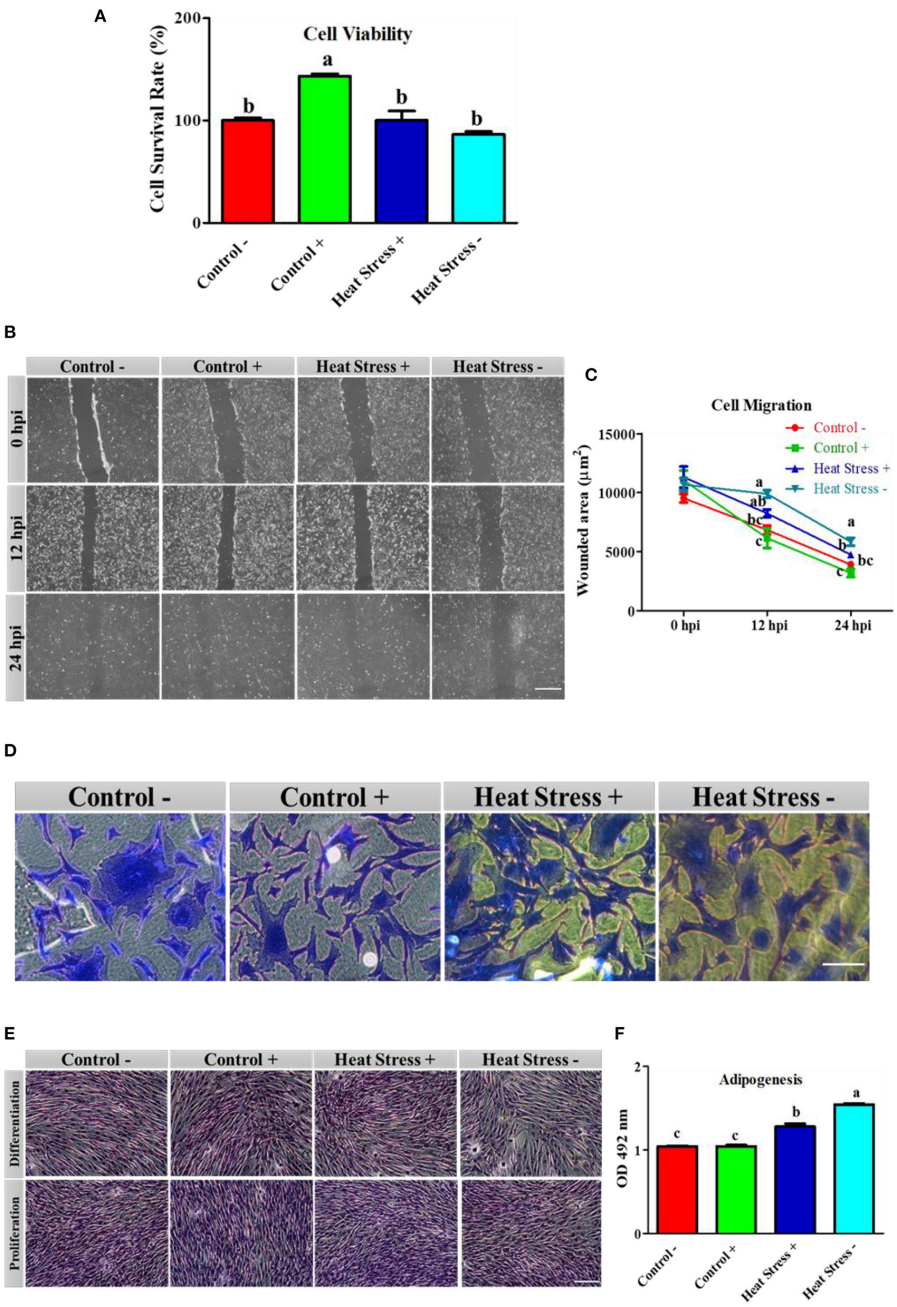
Effects of COPA3 on stressed fibroblasts' viability, cytoskeleton structure, and functions. **(A)** Percentages of fibroblasts' survivability obtained using the CCK-8 assay. **(B)** Fibroblasts' migration activity analysis by wound healing assay; hpi indicates hour post incubation; scale bar = 100 μm. **(C)** Wounded area measurement; data represent mean ±SE; ^a−*c*^different letters indicate significant difference in different groups at specific time periods (*P* < 0.05). **(D)** Coomassie blue stain showing the cytoskeleton shape; scale bar = 20 μm. **(E)** Oil Red O (ORO) staining showing the lipid droplets in fibroblasts; scale bar = 100 μm. **(F)** Adipogenesis in fibroblasts; data represent mean ± SE; ^a−*c*^different letters indicate significant difference in different groups (*P* < 0.05). Groups are distinguished by temperatures and COPA3 treatment, that is, fibroblasts at 38°C (Control- group), 38°C with COPA3 treatment (Control+ group), 43°C with COPA3 treatment (Heat Stress+ group), and 43°C temperature (Heat Stress- group). Each experiment was conducted in four biological replicates (*n* = 4).

To explore how COPA3 affects fibroblast viability and function during heat stress, we analyzed the cell viability in the Control-, Control+, Heat Stress+, and Heat Stress- groups. The cell survivability was significantly higher in the Control+ group and significantly lower in the Heat Stress- group than in the other groups (Figure 2A) as a consequence highest percentage of cells was found in the Control+ group (143.31%), and the lowest percentage of cells was found in the Heat Stress- group (89.38%) (Supplementary Figures 2A, B). Comparisons among groups at different post-incubation time points revealed that the highest degree of cell migration occurred in the Control+ group and the lowest, in the Heat Stress- group (Figure 2B). The wounded area was significantly higher in the Heat Stress group- and was significantly lower in the Control+ group at both 12 h post-incubation (hpi) and 24 hpi compared to the other groups (Figure 2C). The wounded area gradually decreased with incubation time in all the groups (Supplementary Figures 3A–D). To investigate the changes in the fibroblast structure, organization, transmit stress, and shape, we observed the fibroblast cytoskeleton (43). The cell shape was observed to be different in all treatment groups (Figure 2D), suggesting that heat stress and COPA3 affect fibroblast morphology. Notably, heat stress affected the lipid droplets in the cells (Figure 2E). Adipogenesis was significantly higher in both the heat stress groups than in all control groups. Adipogenesis was significantly lower in the COPA3-treated heat stress group than in the Heat Stress- group, although significantly higher in the COPA3-treated heat stress group than in both the control groups (Figure 2F). Higher adipogenesis designates the metabolic disorder (44). This result indicates that heat stress is one of the important factors for abnormal cell metabolism.

#### COPA3 affects cell death through cellular metabolic activity

**Figure 3:**
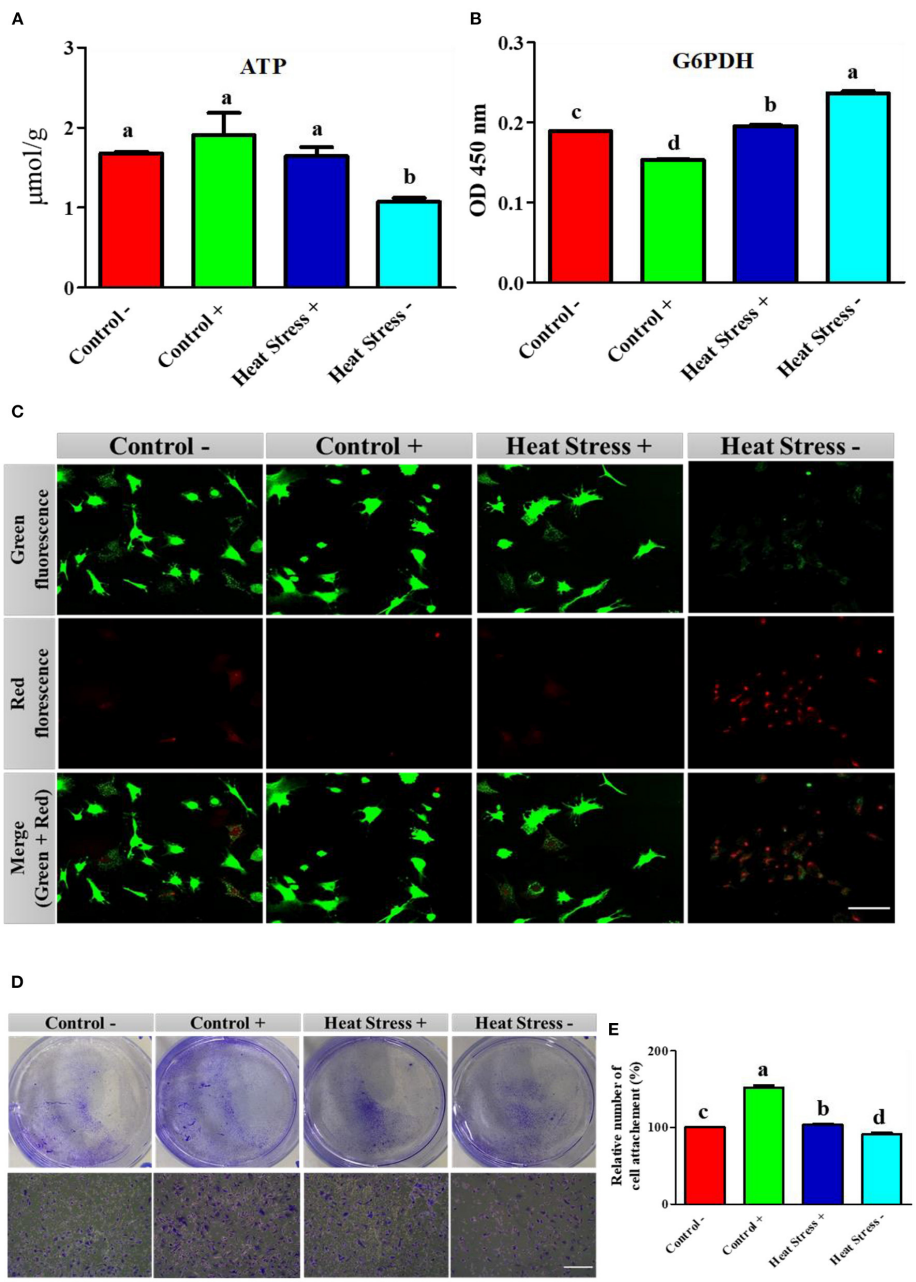
Effects of COPA3 on stressed fibroblasts' apoptosis through energy regulation. **(A)** Concentration of adenosine triphosphate (ATP); data represent mean ± SE; ^a, b^different letters indicate significant difference in different groups (*P* < 0.05). **(B)** Concentration of glucose 6 phosphate dehydrogenase (G6PDH); data represent mean ± SE; ^a, b^different letters indicate significant difference in different groups (*P* < 0.05). **(C)** Live and dead cell stain showing the live cells and apoptosed cells; scale bar = 50 μm. **(D)** Crystal violet stain showing cell attachment; scale bar = 100 μm. **(E)** Percentage of cell attachment; data represent mean ± SE; ^a, d^different letters indicate significant difference in different groups (*P* < 0.05). Groups are distinguished by temperatures and COPA3 treatment, that is, fibroblasts at 38°C (Control- group), 38°C with COPA3 treatment (Control+ group), 43°C with COPA3 treatment (Heat Stress+ group), and 43°C temperature (Heat Stress- group). Each experiment was conducted in four biological replicates (*n* = 4).

To further explore the relationship between adenosine triphosphate (ATP) and cellular activities during heat stress, we analyzed the ATP activity and cell environmental conditions. ATP concentration was significantly decreased in the Heat Stress- group than in the other three groups (Figure 3A). The G6PDH level was significantly higher in the heat stress group. In contrast, G6PDH level was significantly lower in the COPA3-treated control group (Control+) than in the other groups (Figure 3B). Cell viability was assessed by live/dead staining assay after the heat stress and COPA3 treatment of fibroblasts. Cell viability was not found to be affected by COPA3 treatment (Figure 3C). In contrast, the highest number of dead cells was observed in the Heat Stress- group. Fibroblasts were visualized using crystal violet staining (Figure 3D). Heat stress reduced cell attachment and COPA3 was observed to promote cell attachment at 38°C (Figure 3E). These results indicated that COPA3 therapy promoted cell function.

#### COPA3 reduces heat stress level in fibroblasts

**Figure 4:**
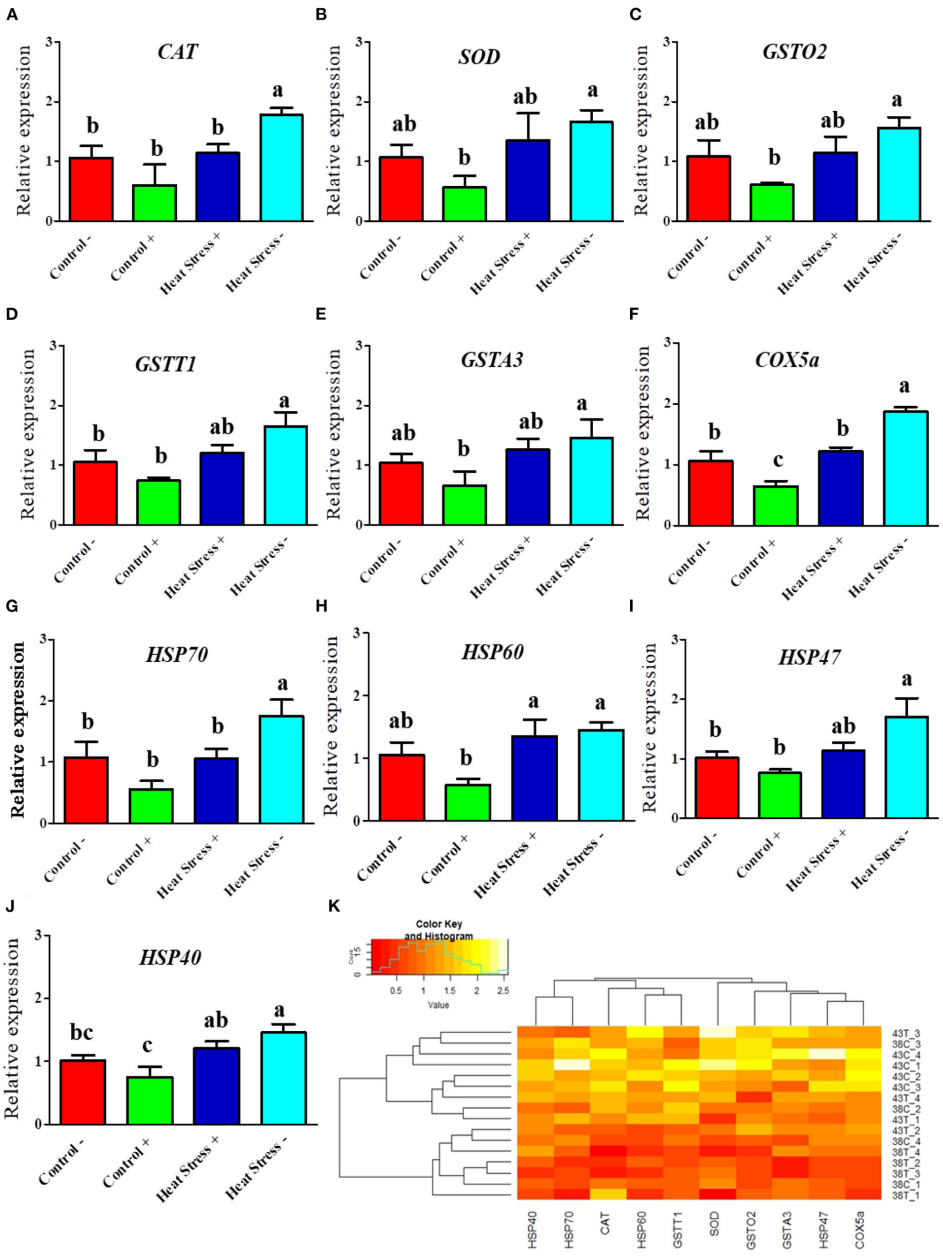
Effects of COPA3 on mRNA expression levels of antioxidant enzymes and heat shock proteins. **(A–J)** Relative mRNA expression levels corresponding to the genes of catalase, superoxide dismutase, glutathione S-transferase omega-2, glutathione S-transferase theta 1, glutathione S-transferase A3, cytochrome c oxidase subunit 5a, heat shock protein70, heat shock protein 60, heat shock protein 47, and heat shock protein 40 obtained by RT-qPCR; data represent mean ± SE; ^a−*c*^different letters indicate significant difference in different groups (*P* < 0.05). **(K)** Cluster analysis of mRNA expression. Groups are distinguished by temperatures and COPA3 treatment, that is, fibroblasts at 38°C (Control- group), 38°C with COPA3 treatment (Control+ group), 43°C with COPA3 treatment (Heat Stress+ group), and 43°C temperature (Heat Stress- group). Each experiment was conducted in four biological replicates (*n* = 4).

To investigate whether the heat stress level was higher in the fibroblasts treated with COPA3, we performed RT-qPCR. The mRNA expression levels of the antioxidant enzymes *CAT, SOD, GSTO2, GSTT1, GSTA3*, and *COX5a* were higher in the Heat Stress- group (Figures 4A–F). In contrast, the mRNA expression level of the antioxidant enzyme *COX5a* was significantly decreased in the COPA3-treated control group. The mRNA expression levels of *HSP70, HSP60, HSP47*, and *HSP40* were higher in the Heat Stress- group (Figures 4G–J) and were lower in the COPA3-treated control group. The mRNA expression levels of the antioxidant enzymes and HSPs were similar in the Control-/+ and Heat Stress+ groups. Clustering analysis of the antioxidant enzymes and heat stress markers in the four different groups showed that the mRNA expression level of the antioxidant enzymes and heat stress markers was higher in the Heat Stress- group than in the other groups (Figure 4K), with no difference observed among the Control-, Control+, and Heat Stress+ groups. This result indicated that COPA3 reduces the heat stress level in fibroblasts.

#### COPA3 modifies the MAPK/ERK-Nrf2 gene expression in heat stressed fibroblasts

**Figure 5:**
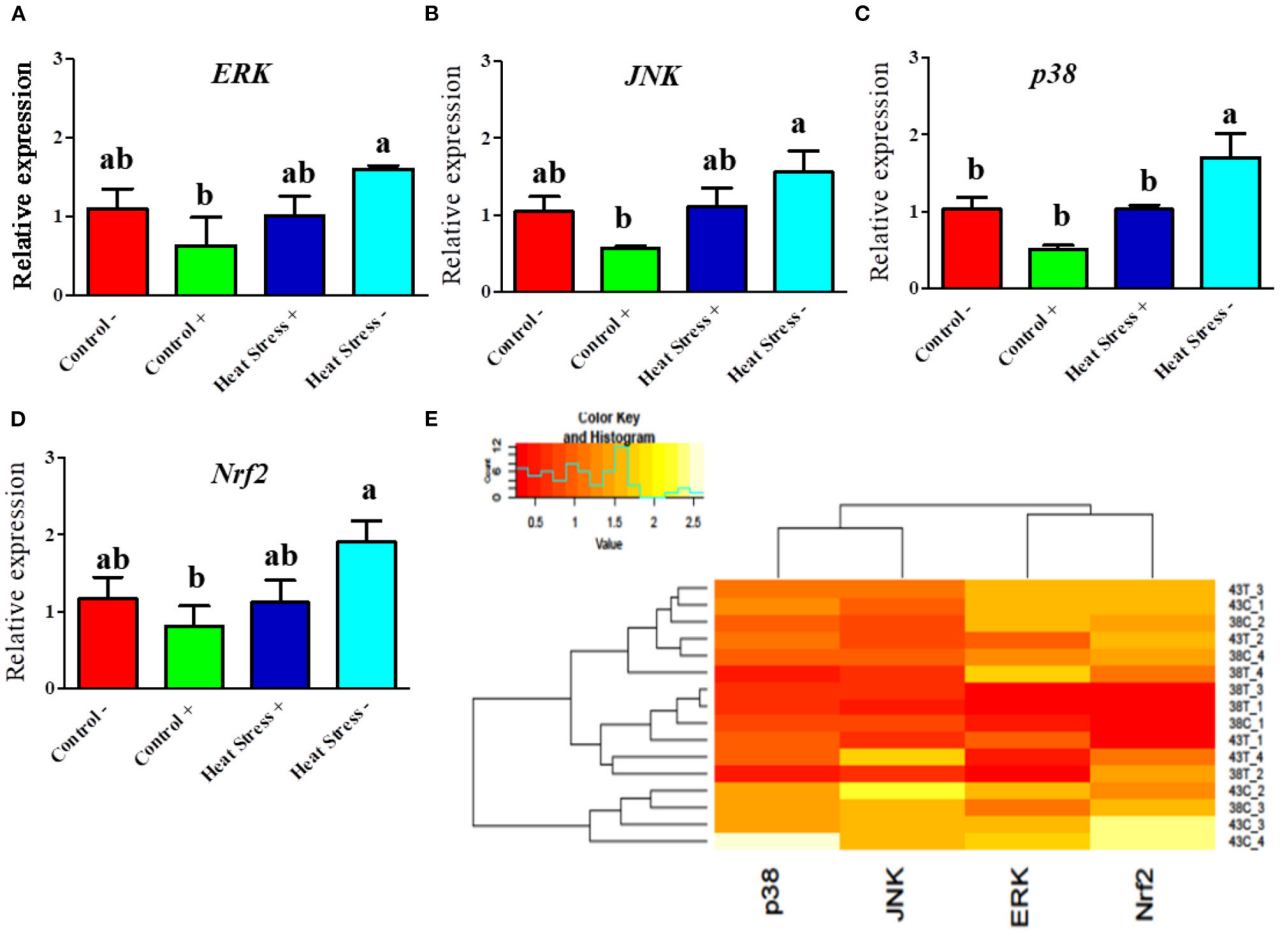
Effects of COPA3 on heat stressed fibroblasts' MAPK/ERK-Nrf2 related gene expression. **(A–D)** Relative mRNA expression levels of the genes of extracellular signal-regulated kinases, jun N-terminal kinase, p38 mitogen-activated protein kinases, and nuclear factor erythroid 2–related factor 2 involved in the MAPK/ERK-Nrf2 obtained by RT-qPCR; data represent mean ± SE; ^a, b^different letters indicate significant difference in different groups (*P* < 0.05). **(E)** Cluster analysis of the mRNA expression corresponding to the MAPK/ERK-Nrf2 components. Groups are distinguished by temperatures and COPA3 treatment, that is, fibroblasts at 38°C (Control- group), 38°C with COPA3 treatment (Control+ group), 43°C with COPA3 treatment (Heat Stress+ group), and 43°C temperature (Heat Stress- group). Each experiment was conducted in four biological replicates (*n* = 4).

Next, we explored whether COPA3 alters the MAPK/ERK-NRF2 gene expression in heat-stressed fibroblasts (Figure 5). Activation of the MAPK is associated with heat stress in cells. *ERK* and *JNK* mRNA expression levels was higher in the Heat Stress- group when compared to the COPA3-treated control group (Control+) (Figures 5A, B). The mRNA expression level of *p38* was higher in the Heat Stress- group compared to the other groups (Figure 5C). With high heat stress level, mRNA expression level of *Nrf2* significantly increased in Heat Stress- group compared to the other groups, whereas the mRNA expression level of *Nrf2* was statistically similar between the Control-, Control+, and Heat Stress+ groups (Figure 5D). Clustering analysis of the MAPK/ERK and Nrf2 gene expression in the four different groups indicated that the Heat Stress-treated group was distinct from the other groups (Figure 5E). However, COPA3 promoted cell proliferation in both heat-stress and control temperatures. This result suggested that cell proliferation and differentiation were stalled in Heat Stress- group caused of stress temperature. COPA3 induced cell proliferation by maintaining the stress temperature.

#### Correlation and characterization of heat stress and COPA3 treatment in fibroblasts

**Figure 6:**
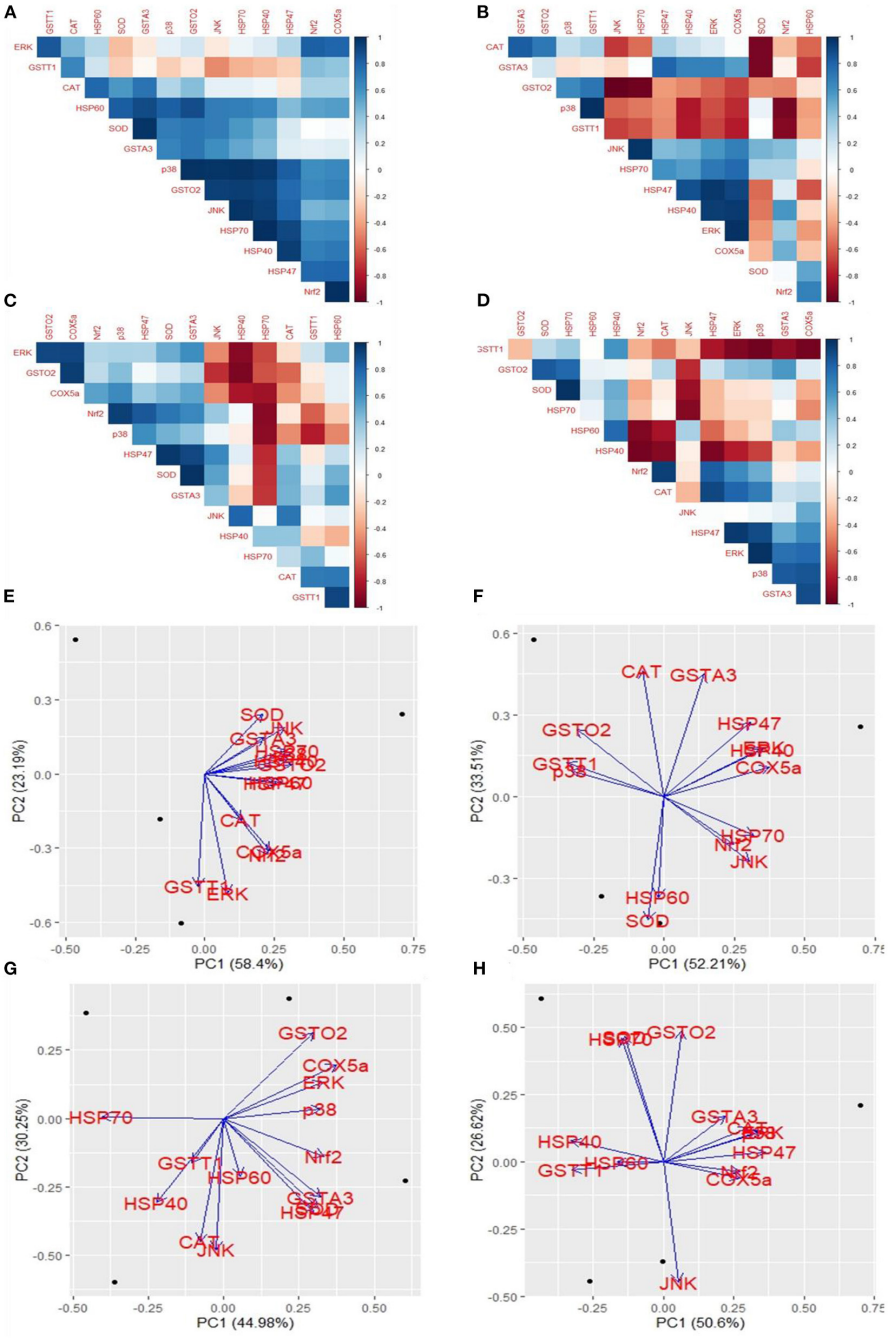
Effects of COPA3 on the relationship between heat and MAPK/ERK-Nrf2 mRNA expression in fibroblasts. Correlations among the mRNA expression levels of the antioxidant enzymes, heat shock proteins, and the MAPK/ERK-Nrf2 related gene. **(A)** Control- group, **(B)** Control+ group, **(C)** Heat Stress+ group, and **(D)** Heat Stress- group. Principal component analysis (PCA) of the mRNA expression levels of the antioxidant enzymes, heat shock proteins, and the MAPK/ERK-Nrf2 related gene. Based on the position of the different genes on the X and Y axis predict the relation among different genes in particular group. **(E)** Control- group, **(F)** Control+ group, **(G)** Heat Stress+ group, and **(H)** Heat Stress- group. PC1 and PC2 were selected as they explained the most variation.

The cross-platform of mRNA expression data is challenging. A treatment that modifies gene expression in one group did not apply to another group. We used the coefficient correlation method to minimize the sample and platform variations. It compares the transcriptome-wide correlation between two different genes of specific group by analyzing the vector of coefficient correlation and then analyzes the coefficient correlation between the two recommended vectors. In this study, we analyzed the correlation between 14 mRNA expression levels in four fibroblast groups. In the control group, *HSP60* and *SOD* were positively correlated with *GSTA3*; *p38* was positively correlated with *GSTO2, JNK, HSP70*, and *HSP40; GSTO2* was positively correlated with *JNK, HSP70*, and *HSP47; JNK* was positively correlated with *HSP70* and *HSP40*; and *Nrf2* was positively correlated with *COX5a* (Figure 6A). In the Control+ group, *CAT* was positively correlated with *GSTA3*; *p38* was positively correlated with *GSTT1*; *JNK* was positively correlated with *HSP70*; *ERK* was positively correlated with *HSP47* and *HSP40*; and *COX5a* positively correlated with *HSP40* and *ERK*; contrastingly, *GSTO2* was negatively correlated with *JNK* and *HSP70*; *SOD* was negatively correlated with *GSTA3* and *CAT*; and *Nrf2* was negatively correlated with *p38* and *GSTT1* (Figure 6B). In the Heat Stress+ group, *ERK* was positively correlated with *GSTO2* and *COX5a; GSTO2* was positively correlated with *COX5a; Nrf2* was positively correlated with *p38; SOD* was positively correlated with *HSP47*; *GSTA3* was positively correlated with *HSP47* and *SOD*; and *GSTT1* was positively correlated with *HSP60*; contrastingly, *HSP40* was negatively correlated with *ERK, GSTO2*, and *COX5a*; and *HSP70* was negatively correlated with *COX5a, Nrf2*, and *p38* (Figure 6C). In the Heat Stress- group, *SOD* was positively correlated with *HSP70*; and *ERK* was positively correlated with *HSP47* and *p38*; contrastingly, *Nrf2* was negatively correlated with *HSP60* and *HSP40*; *JNK* was negatively correlated with *SOD* and *HSP70; HSP47* was negatively correlated with *HSP40*; and *GSTT1* was negatively correlated with *ERK, p38*, and *COX5a* (Figure 6D). Eventually, heat stress and control were positively correlated (*R* = 0.49) (Supplementary Figure 4). This result indicated that COPA3 modifies the relationship between heat stress and the MAPK/ERK-Nrf2 gene expression.

We performed a principal component (PC) analysis on the 14 parameters to investigate the association between heat stress and the MAPK/ERK-Nrf2 gene expression. This association was compared among all genes of our study, with respect to the Control-, Control+, Heat Stress-, and Heat Stress+ groups. In the control group, the first two principal components explained a total variance of 81.59% among the 14 analyzed genes (PC1 = 58.4% and PC2 = 23.19%); heat stress and the MAPK/ERK-Nrf2 gene expression were positively associated (Figure 6E). In the Control+ group, the first two principal components explained 85.72% of the total variance among the 14 analyzed genes (PC1 = 52.21% and PC2 = 33.51%); *HSP60* and *SOD* were negatively associated with *GSTA3, HSP47, HSP40, ERK*, as well as *COX5a; JNK, Nrf2*, and *HSP70* were negatively associated with *CAT, GSTO2, GSTT1*, and *p38* (Figure 6F). In Heat Stress+ group, the first two principal components explained 75.23% of the total variance among the 14 analyzed genes (PC1 = 44.98% and PC2 = 30.25%); *GSTT1, HSP40*, and *CAT* were negatively associated with *ERK, COX5a*, and *GSTO2* (Figure 6G). In the heat stress group-, the first two principal components explained 77.22% of the total variance among the 14 analyzed genes (PC1 = 50.6% and PC2 = 26.62%); *JNK* was negatively associated with *HSP40, SOD, HSP70*, and *GSTO2* (Figure 6H). Clustering analysis of different groups indicated that the gene expression levels differed among groups (Supplementary Figure 5). We analyzed the chord diagram to examine the interrelationship between the data and matrix. Our results showed that gene expression data were related to the Heat Stress- group (Supplementary Figure 6). These results suggested that COPA3 promoted proliferation and differentiation in fibroblasts by reducing the severity of heat stress through MAPK/ERK-Nrf2 related genes regulation.

## Discussion

Heat stress, an important risk factor for cell death, affects cellular proliferation, damages cellular organelles, and disrupts organelle functions (5, 45, 46). Antioxidants enable cells to withstand or suppress the harmful effects caused by free radicals, especially ROS, on cells (47). Moreover, during heat stress, antioxidants protect the mitochondrial functions and repair the mitochondrial electron transport chain (ETC) by reducing free radical production and converting toxic free radicals to nontoxic elements (48). In the present study, we investigated the potential of COPA3 to be used as an antioxidant by investigating the MAPK/ERK-Nrf2 gene expression. Our findings imply that COPA3 maintains the proliferation and differentiation of fibroblasts by showing anti-stress activity like antioxidants, under heat-stress conditions. In our study, COPA3 showed strong anti-stress activity by maintaining the homeostasis of cell morphology and function. Our results revealed that COPA3 maintains stress levels and fibroblast morphology.

In biological systems, heat stress induces alterations in cell shape, cell structure cytoskeleton, and functions (49, 50). Previous studies have reported that peptides may exhibit different biological functions simultaneously (51, 52). On the other side, heat stress reduces cell viability and proliferation (53). Fortunately, the COPA3 increases cell proliferation by preventing inflammation (33). This corresponds to our study result that COPA3 possesses heat stress scavenging ability. Moreover, our results revealed that COPA3 increases fibroblast migration activity, maintains homeostasis of the cytoskeleton structure, and inhibits adipogenesis. In contrast, heat stress enhances adipogenesis in porcine stromal vascular cells adipocytes (54). Stress is a major factor in cell death, leading to decreased cell attachment and colony formation (55, 56). Heat stress also prevents the production of energy, a key factor for cell survival (57), by damaging the mitochondria (58). This is consistent with our study results that showed that ATP level decreased upon heat stress (1), eventually inducing apoptosis and decreasing cell attachment. Contrastingly, COPA3 induces cell survival and cell attachment by homeostasis the energy level.

The function and structure of the stressed cells vary with the stress level. Heat stress level can be measured using different antioxidant enzymes and heat stress marker expression levels (59). Heat stress induces cell damage by degrading the cellular defense mechanism. Antioxidant enzymes catalyze the transformation of ROS, thus reducing the Heat Stress- induced cell damage by enhancing the defense mechanism (60). Therefore, the gene expression levels of antioxidant enzymes are elevated during heat stress. Previous studies have reported that heat stress increases antioxidant activity and gene expression to promote heat tolerance (61–63). HSP levels increase during heat stress environmental conditions to repair the misfolded proteins, in order to protect cells (63, 64). Our results demonstrated that heat stress increased the expression levels of antioxidant enzymes and HSPs, whereas the gene expression levels of antioxidant enzymes and HSPs did not change significantly after COPA3 treatment. As expected, heat stress maintains the homeostasis of cell proliferation and differentiation by triggering the MAPK/ERK gene expression (23, 65). A high expression level of Nrf2 plays a protective role against Heat Stress- induced apoptosis (66). Our results revealed that COPA3 treatment maintains the MAPK, ERK, and Nrf2 gene expression under heat stress conditions same as the control group and implied that antioxidant properties are exhibited by COPA3, which maintains fibroblast proliferation and differentiation during heat stress (Supplementary Figure 7).

## Conclusion

Our work highlights that COPA3 peptide exerts its antioxidant effects by altering the MAPK/ERK-Nrf2 related gene expression in heat-stressed fibroblasts, and it increases the proliferation and differentiation of fibroblasts at favorable temperature as well as prevent the protein from misfolding. The specific site of action of this peptide remains to be investigated, and further studies are needed to identify the COPA3 peptide responsible for the antioxidant activity by using sequencing. COPA3 exerts antioxidant activity by maintaining fibroblasts proliferation and differentiation. Further study needs to explore the COPA3 binding site, which will help to find out the cellular functions during COPA3 treatment on cells, and confirmation the function of COPA3 on cellular activity.

## Data availability statement

The datasets presented in this study can be found in online repositories. The names of the repository/repositories and accession number(s) can be found in the article/Supplementary material.

## Ethics statement

All experiments in our study were performed according to the relevant rules and regulations of the Jeonbuk National University. All animal care protocols and experimental protocol were approved and supervised by the Animal Experiment Administration Committee of our university (Approval Number: CBNU 2019–020).

## Author contributions

Conceived and designed the experiments: SS, KS, MK, and DK. Performed the experiments, prepared the samples and analyzed the data, and wrote the manuscript: SS and MK. Revised the manuscript: JP, JL, DK, HC, and KS. Obtained the finance support: KS and DK. All authors read and approved the final manuscript.
