# Supplementary material for: COPA3 peptide supplementation alleviates the heat stress of chicken fibroblasts
Source: Front Vet Sci. 2023 Feb 24;10:985040. doi: 10.3389/fvets.2023.985040 (PMC9998527; doi:10.3389/fvets.2023.985040)
Supplement: Supplementary file 1 [file Data_Sheet_1.docx]

Supplementary Material

**Supplementary Figures**


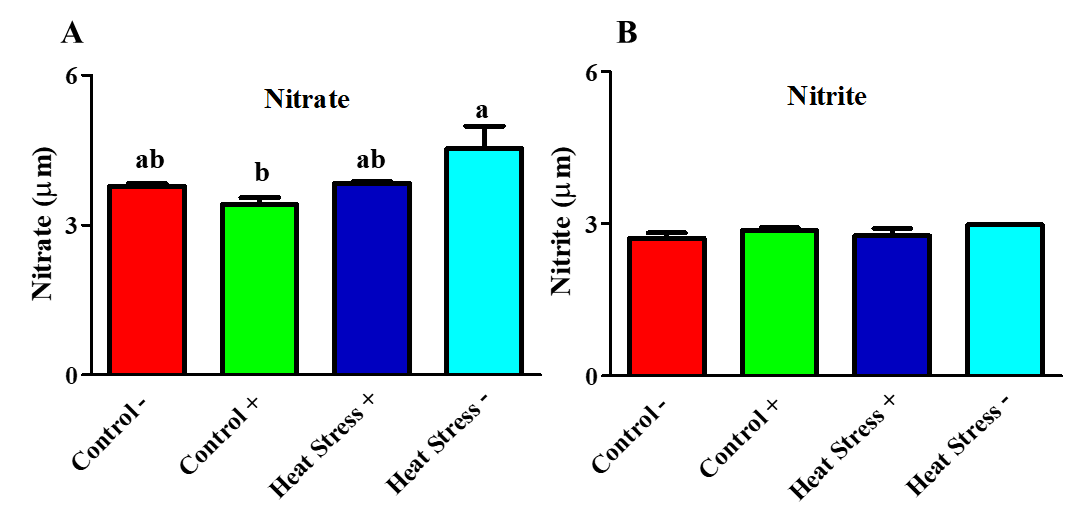


**Supplementary Figure 1. Effect of COPA3 on the amount of nitrate and nitrite synthesized in stressed fibroblasts**. **A** Concentration of nitrate; data represent mean ± SE; ^a-b^ different letters indicate significant difference in different groups (*P* < 0.05). **B** Concentration of nitrite; data represent mean ± SE. Groups are distinguished by temperatures and COPA3 treatment, that is, fibroblasts at 38°C (Control- group), 38°C with COPA3 treatment (Control+ group), 43°C with COPA3 treatment (Heat Stress+ group), and 43°C temperature (Heat Stress- group). Each experiment was conducted in four biological replicates (n = 4).


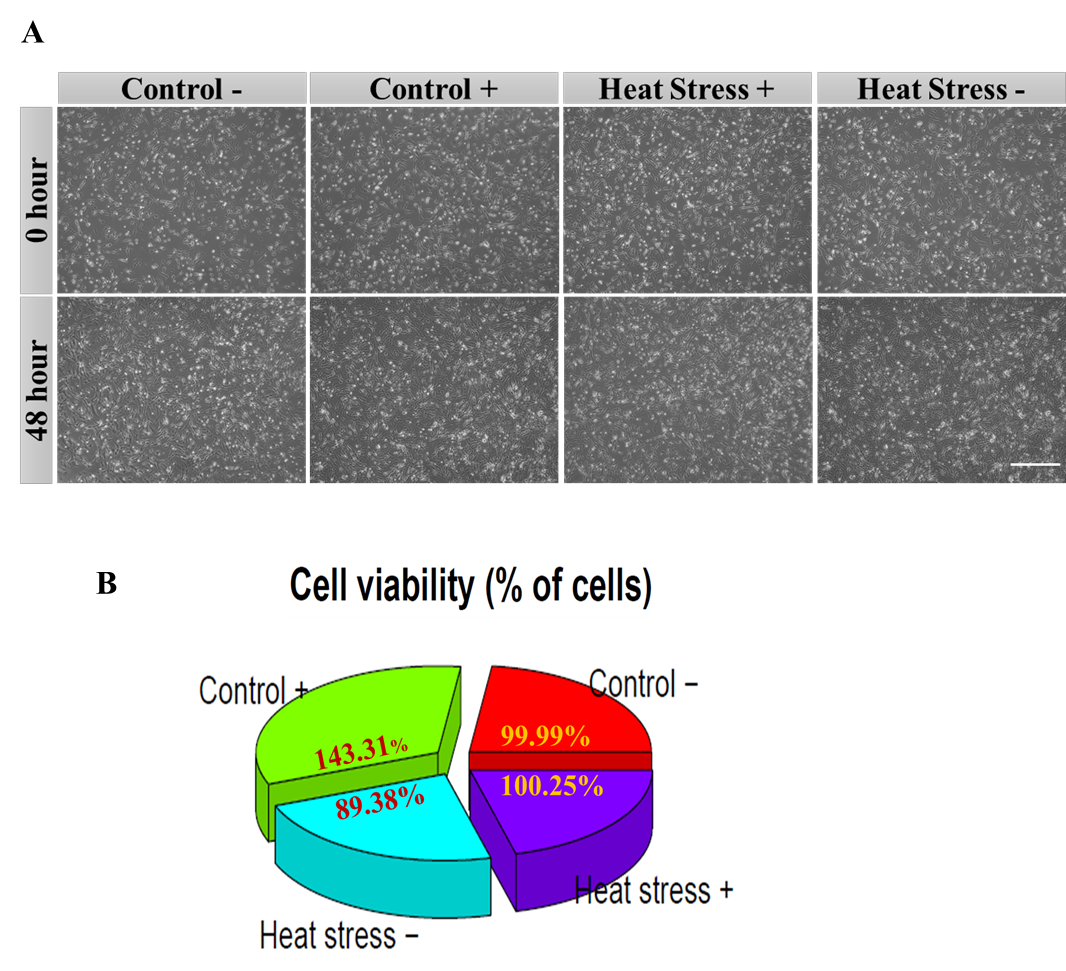


**Supplementary Figure 2. Effect of COPA3 on fibroblasts' survival rate. a** Microscopic image of the viable fibroblasts; scale bar = 50 µm. **b** Percentage of fibroblasts survivability obtained using the CCK-8 assay; data represent mean ± SE; ^a-c^ different letters indicate significant difference in different groups (*P* < 0.05). Groups are distinguished by temperatures and COPA3 treatment, that is, fibroblasts at 38°C (Control- group), 38°C with COPA3 treatment (Control+ group), 43°C with COPA3 treatment (Heat Stress+ group), and 43°C temperature (Heat Stress- group). Each experiment was conducted in four biological replicates (n = 4).


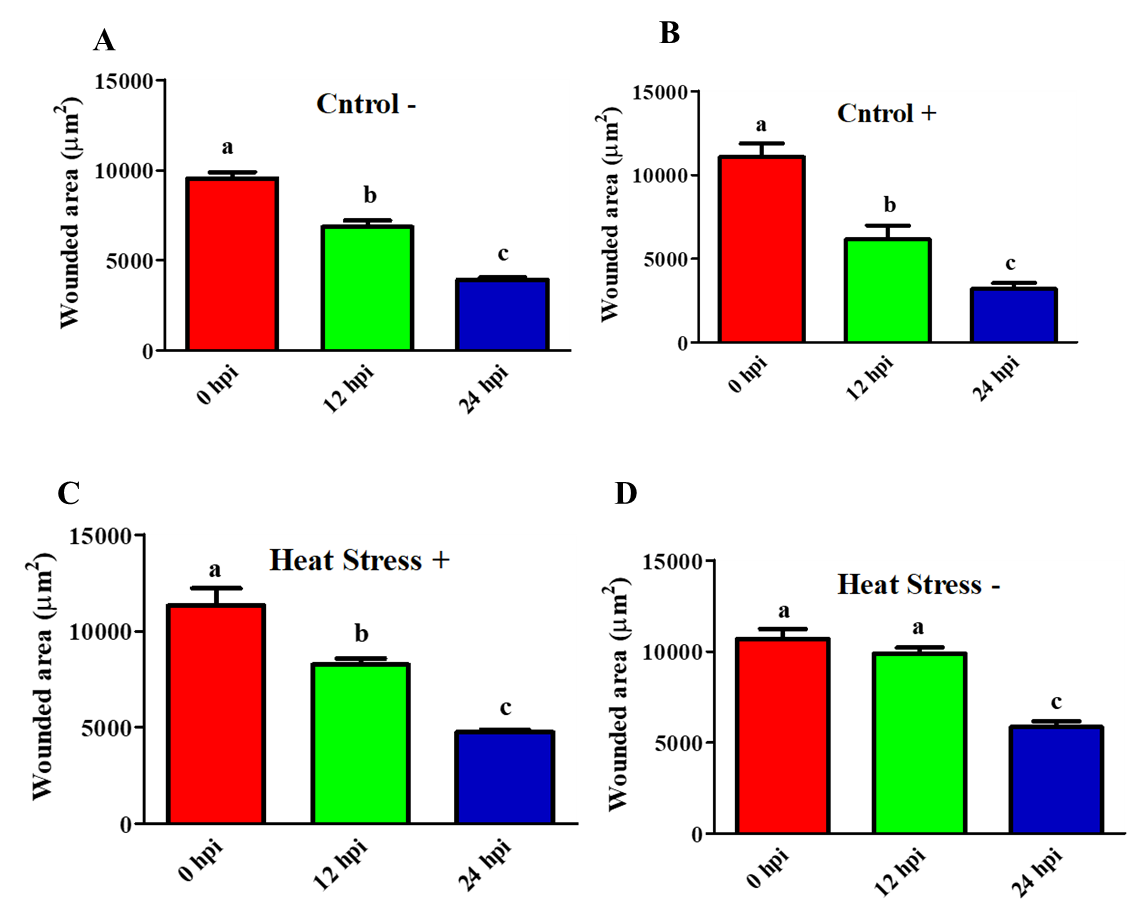


**Supplementary Figure 3. COPA3 accelerates the fibroblasts' migration process. A-D** Total wounded area after treatment of the fibroblasts with heat stress and COPA3; data represent mean ± SE; ^a-c^ different letters indicate significant difference in different groups (*P* < 0.05). Groups are distinguished by temperatures and COPA3 treatment, that is, fibroblasts at 38°C (Control- group), 38°C with COPA3 treatment (Control+ group), 43°C with COPA3 treatment (Heat Stress+ group), and 43°C temperature (Heat Stress- group). Each experiment was conducted in four biological replicates (n = 4).


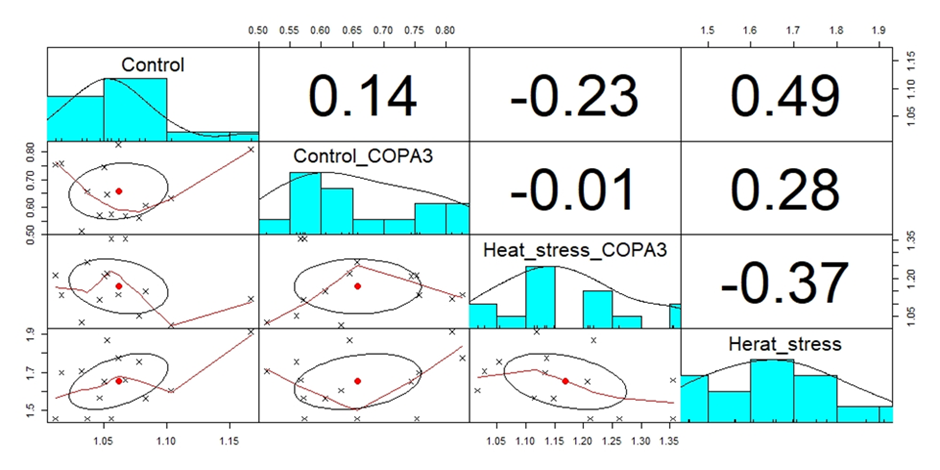


**Supplementary Figure 4. Correlation among different groups of heat stress and COPA3 treatment**. Groups are distinguished by temperatures and COPA3 treatment, that is, fibroblasts at 38°C (Control- group), 38°C with COPA3 treatment (Control+ group), 43°C with COPA3 treatment (Heat Stress+ group), and 43°C temperature (Heat Stress- group).


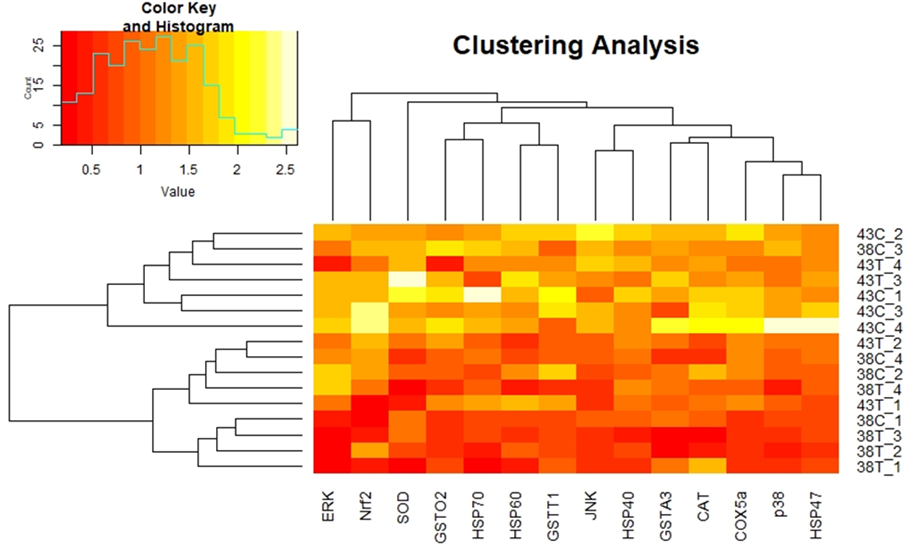


**Supplementary Figure 5. Clustering analysis of mRNA expression levels.** Groups are distinguished by temperatures and COPA3 treatment, that is, fibroblasts at 38°C (Control- group), 38°C with COPA3 treatment (Control+ group), 43°C with COPA3 treatment (Heat Stress+ group), and 43°C temperature (Heat Stress- group). Each experiment was conducted in four replicates (n = 4).


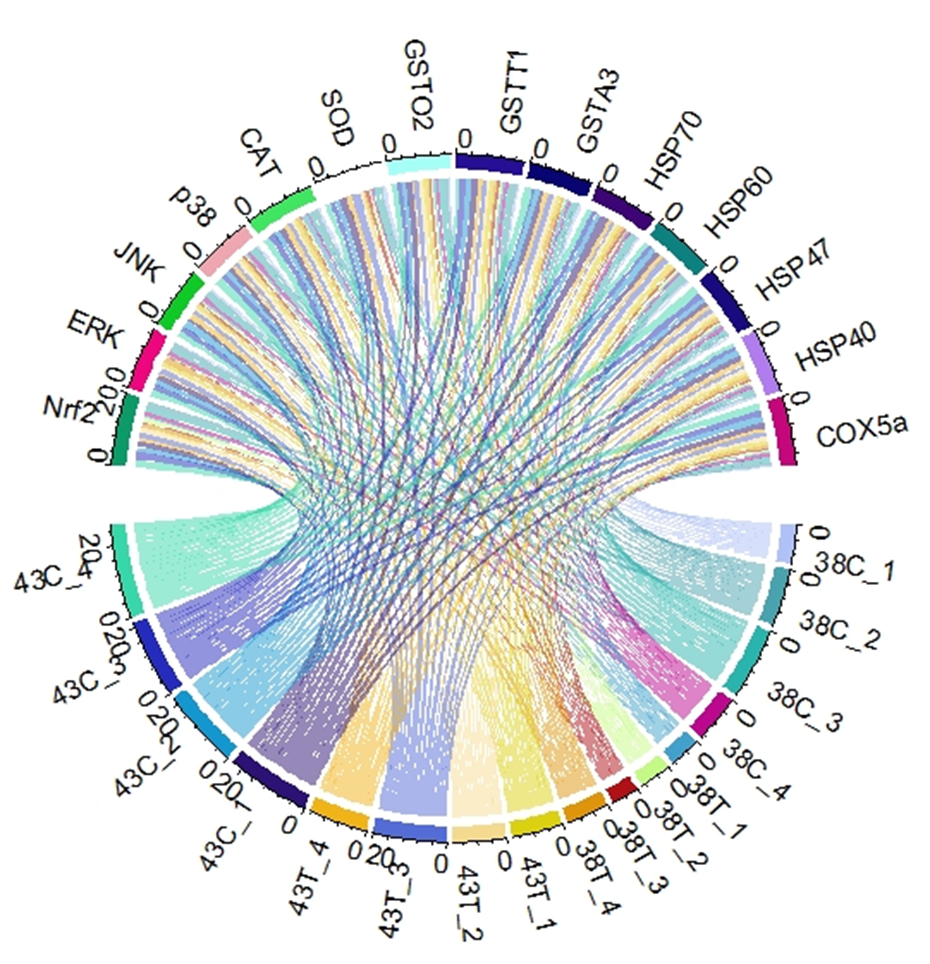


**Supplementary Figure 6. The chord diagram represents an association between MAPK/ERK-Nrf2 pathways and heat stress levels' genes.** The data are arranged radially around a circle showing the relationships between the data points. Groups are distinguished by temperatures and COPA3 treatment, that is, fibroblasts at 38°C (Control-), 38°C with COPA3 treatment (Control+), 43°C with COPA3 treatment (Heat Stress+), and 43°C temperature (Heat Stress-).


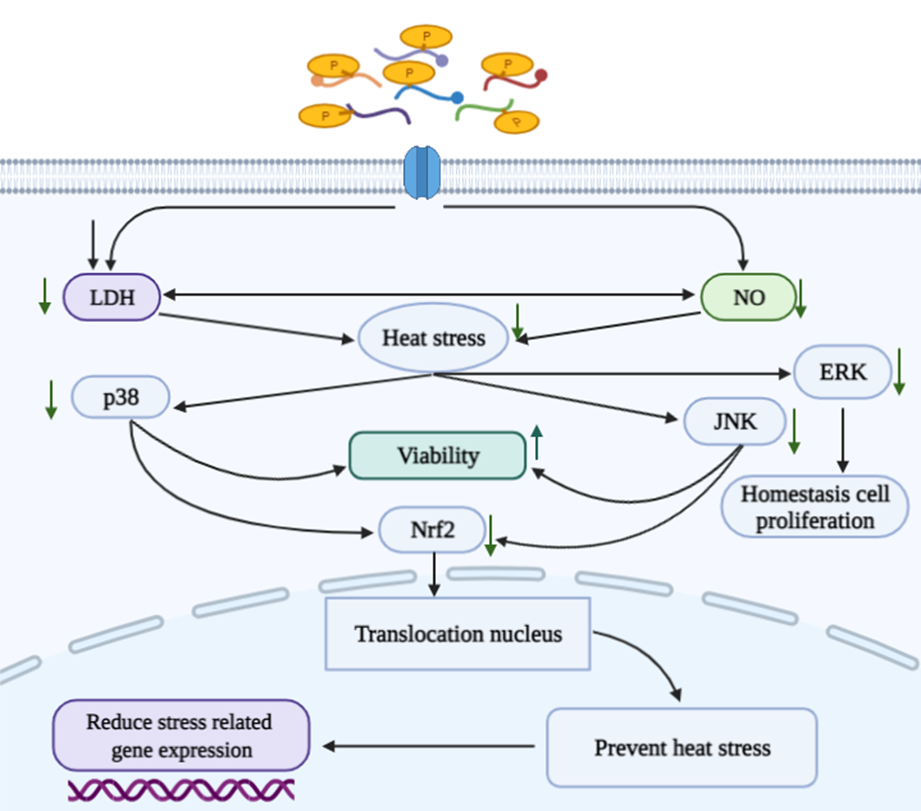


**Supplementary Figure 7.** **Graphical description of COPA3 reduces the heat stress level on fibroblasts**.

**Table Supplementary 1.** Primer sequences, amplicon size of different gene for RT-qPCR analysis.

| **Gene symbol** | **Accession number** | **Primer sequences (5-3)** | **Amplicon size (bp)** | **TM(°C)** |
| --- | --- | --- | --- | --- |
| CAT | NM_001031215.2 | F- ACCAAGTACTGCAAGGCGAAAGT  R- ACCCAGATTCTCCAGCAACAGTG | 91 | 62 |
| SOD | NM_205064.1 | F-TTGTCTGATGGAGATCATGGCTTC  R-TGCTTGCCTTCAGGATTAAAGTGAG | 98 | 60 |
| GSTO2 | XM_015288649.3 | F-CATGATGTGGCCCTGGTTTG  R-CAGTGCTGGAGCTTTGGAGTATGA | 101 | 60 |
| GSTT1 | NM_205365.1 | F-GACGGAGACTTCACCCTAGCAGA  R-TGATGGGTACCAGTGGTCAGGA | 87 | 62 |
| GSTA3 | NM_001001777.1 | F-TTGGATAAGGCCGCAAACAGATA  R-TTTCCAGTAAATGCACGTCTGCTC | 115 | 60 |
| Nrf2 | XM_015289387.3 | F- ATC ACG AGC CCT GAA ACC AA  R- GGC TGC AAA ATG CTG GAA AA | 143 | 60 |
| ERK | XM_015275131.3 | F- AGC AAG CTT TAG CCC ATC CA  R- CCT TCG GCA AGT CAT CCA AT | 108 | 59 |
| JNK | NM_205095.1 | F- AGC AGC CTC GAT GCC TTG AC  R- CAA GCA ATT CAG GCC CAA TG | 110 | 60 |
| p38 | XM_040691291.1 | F- TGT GTT CAC CCC TGC CAA GT  R- GCC CCC GAA GAA TCT GGT AT | 149 | 60 |
| HSP70 | NM_001006685.1 | F-GGTAAGCACAAGCGTGACAATGCT  R-TCAATCTCAATGCTGGCTTGCGTG | 116 | 64 |
| HSP60 | NM_001012916.2 | F-AGAAGAAGGACAGAGTTACC  R-GCGTCTAATGCTGGAATG | 112 | 54 |
| HSP47 | XM_040665757.1 | F-ACTGGCTCATAAGCTCTCCAGCAT  R-TCATCTTGCTGGCCCA GGTCTTTA | 116 | 64 |
| HSP40 | XM_015275726.3 | F-GGGCATTCAACAGCATAGA  R-TTCACATCCCCAAGTTTAGG | 151 | 55 |
| COX5a | XM_040680176.1 | F-CTCCACTCCAGAGGAACTGG  R-TCTGACTTGAGGCGACTGTG | 106 | 59 |
| GAPDH | NM_204305.1 | F-AGAACATCATCCCAGCGTCC  R-CGGCAGGTCAGGTCAACAAC | 133 | 60 |

**Abbreviations**: *CAT, catalase; SOD, Superoxide dismutase; GSTO2, Glutathione S-transferase omega-2; GSTT1, glutathione S-transferase theta 1; GSTA3, Glutathione S-transferase A3; nuclear factor erythroid 2–related factor 2; ERK, extracellular signal-regulated kinases; Jun N-terminal kinase; P38, p38 mitogen-activated protein kinases; HSP70, heat shock protein; HSP60, heat shock protein; HSP47; heat shock protein 47; HSP40, heat shock protein 40, COX5a, Cytochrome c oxidase subunit 5a; GAPDH, Glyceraldehyde-3-Phosphate Dehydrogenase.*
